# Upregulation of EphA4 deteriorate brain damage by shifting microglia M1-polarization via NF-κB signaling after focal cerebral ischemia in rats

**DOI:** 10.1016/j.heliyon.2023.e18429

**Published:** 2023-07-19

**Authors:** Hui-Xing Wei, Yun-Ni Guan, Ping-Ping Chen, Zhao-Zeng Rao, Jin-Shan Yang

**Affiliations:** a Department of Neurology, The First Affiliated Hospital of Fujian Medical University, Fuzhou, PR China; b Fujian Key Laboratory of Molecular Neurology, Fujian Medical University, Fuzhou, PR China; c Department of Neurology, Xiang'an Hospital of Xiamen University, Xiamen, PR China; d Department of Neurology, Longyan People's Hospital, Longyan, PR China; e Department of Neurology, National Regional Medical Center, Binhai Campus of the First Affiliated Hospital, PR China

**Keywords:** Ischemic stroke, Post-stroke inflammation, Neuronal EphA4, Microglia polarization, NF-κB signaling

## Abstract

Ischemic stroke is the main reason of disability and mortality in many countries, and currently has limited treatments. The post-stroke inflammation characterized with microglia activation and polarization has been regarded as a promising therapeutic target for ischemic stroke. After ischemia, the activated microglia polarize to classical (M1) phenotype or alternative (M2) phenotype and exhibit biphasic function. Promoting microglia phenotype shift from deleterious M1 phenotype to neuroprotective M2 phenotype will be promising in stroke treatment. Increasing evidence indicates that the erythropoietin-producing human hepatocellular (Eph) receptor A4 (EphA4), a kind of abundant Eph receptor, distributes mainly in neuron and participates in multiple links of pathological changes after ischemia. This paper discussed the hypothesis that EphA4 receptor could affect ischemic brain injury through EphA4/ephrin bidirectional signaling between neuron and microglia, and then explored its underlying mechanisms. We manipulated EphA4/ephrin signaling with either EphA4 overexpression lentiviral vectors or the short hairpin RNA (shRNA) to upregulate or knock down neuronal EphA4 expression. NF-κB inhibitor pyrrolidine dithiocarbamate ammonium salt (PDTC) was applied to block NF-κB pathway. According to the experimental results, upregulated neuronal EphA4 induced by ischemia deteriorated neurological function as well as brain damage by shifting microglia M1-polarization via promoting NF-κB signaling.

## Graphical abstract

**Figure:**
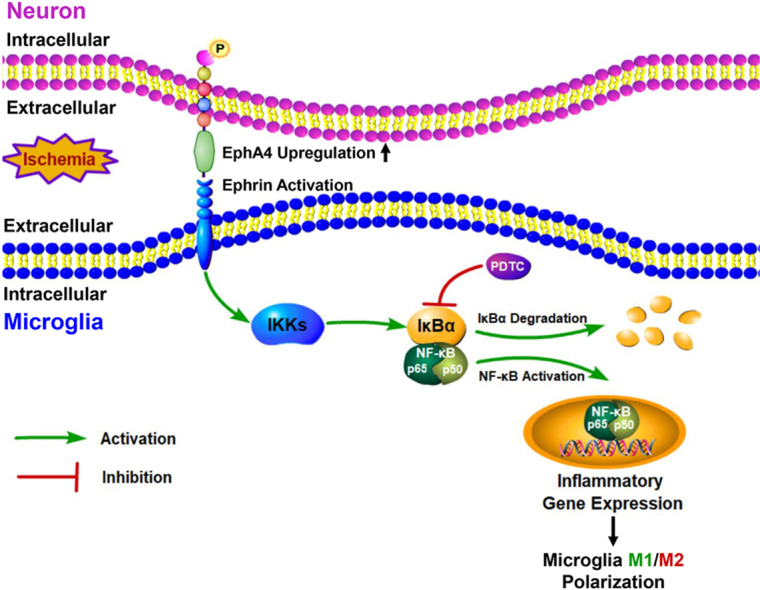

## 1 Introduction

Ischemic stroke (IS), is very closely related to long-term disability and mortality all over the world. Recanalization treatment including thrombolysis and embolectomy is the first choice of treatment for IS, but is restricted by its narrow time window. Post-stroke inflammation, characterized with microglia activation and circulating immune cells invasion, initiates during the early stage of IS and turns out to be the predominant mechanism of ischemia-induced injury lasting for several days. Until recently, the post-stroke inflammatory injury has emerged as new target for IS treatment [1].

Microglia are the resident immune cells of the central nervous system (CNS), and act as the front line of defense against CNS disorders such as ischemic stroke. After ischemia, the activated microglia may polarize to two distinct phenotypic types: M1 or M2 phenotype. Microglia of different phenotype exhibit a double-edged sword effect. M1-phenotype microglia mainly releases cytotoxic substances, induces inflammation, and leads to brain damage. Whereas, M2-phenotype microglia engulfs cell debris, releases neurotrophic factor and shows neuroprotective effect on the brain. As consequence, balancing microglia M1/M2 polarization is promising in stroke treatment [2,3].

Eph receptors compose the biggest receptor tyrosine protein kinase (RTPK) family. The Eph receptors, in combination with their membrane-combined ephrin ligands, constitute a bidirectional signaling. Eph/ephrin signaling acts simultaneously on Eph receptor-expressing as well as ephrin ligand-expressing cells [4]. EphA4 receptor, the most abundant Eph receptor in CNS, is mostly expressed in neurons. The relative previous findings suggested that neuronal EphA4 receptor upregulated and was related to the several pathophysiologic process of IS [[4], [5], [6]]. EphA4/ephrin bidirectional signaling affects the way neuron communicate with glia cells and therefore regulate ischemic brain injury. However, the precise mechanisms need to be further studied.

Accumulating evidence suggest that EphA4 participate in neuroinflammation and tissue damage in spinal cord injury [7] and traumatic brain injury [8], but due to the influence of many factors, its exact pathogenesis is yet unclear. Furthermore, recent studies have implicated that a small molecule EphA4 inhibitor may exert its beneficial effects through modulating microglia function via NF-κB signaling [8].

In this article, the impact of neuronal EphA4 in post-stroke inflammation was discussed. The EphA4 overexpression (EphA4-OE) and EphA4 shRNA (sh-EphA4) lentiviral vectors was used to regulate EphA4/ephrin signaling. The function of EphA4 receptor in post-stroke inflammation was studied in vivo and in vitro by testing the changes in neurological deficit, brain damage, microglial function and NF-κB signaling. We found that EphA4/ephrin bidirectional signaling and downstream NF-κB altered microglial phenotype, and had a further impact on ischemia-induced neurological impairment and brain damage.

## 2 Materials and methods

### 2.1 Construction of EphA4 overexpression and EphA4 shRNA lentiviral vectors

To construct the EphA4 overexpression (EphA4-OE) lentiviral vector, full length of mouse Epha4 cDNA with sequences matching NCBI RefSeq sequences NM_007936 was cloned into FV040 plasmid between the pCMV and the EGFP reporter gene following the manufacturer's instruction. Positive clones were confirmed by RT-PCR method. For plasmid amplification, we transformed the positive plasmid into *Escherichia coli* DH5α cells (TransGen Biotech, China). EphA4-OE plasmids were next transfected into 293T cells for lentivirus production (System Biosciences, USA). At day in vitro (DIV) 2, primary neurons were transfected by lentiviral vectors with the viral titer 1 × 10^8^ TU/ml for 24 h. Primary neurons were cultured for another 2 d [9]. The transfected neurons were indicated by green fluorescence protein (GFP). The overexpression efficiency estimated on mRNA level was detected by RT-PCR technology, and target protein expression was estimated using Western Blot (WB).

Three sh-EphA4 candidates had been constructed and screened in our previous study. The most efficient silencing sequence of sh-EphA4 was selected out for this research. The shRNA sequence was as follow: sense (5′-3′) GATCCGGAAGAATGATGGCCGCTTTACTCGAGTA

AAGCGGCCATCATTCTTCCTTTTTG; Anti-sense (5′-3′) AATTCAAAAAGGAAGAATG

ATGGCCGCTTTACTCGAGTAAAGCGGCCATCATTCTTCCG. The nonspecific sequence 5′-ATGATGGCCGCTTTACTCG-3′ was applied as negative control [6]. EphA4 shRNA lentiviral vectors were cloned into the pGreenPuro vector (System Biosciences, USA) in accordance with the manufacturer's protocol. Briefly, the pGreenPuro™ vector was first linearized with EcoRI/BamHI. Then the shRNA Template Oligonucleotides were annealed at 95 °C for 5 min, 85 °C for 5 min, at 75 °C for 5 min, at 70 °C for 5 min and cooled to room temperature. The shRNA template was further ligated into linearized pGreenPuro™ lentivector. Positive plasmid amplification, lentivirus production, neuron transfection and analysis of silencing efficiency were carried out as described before.

### 2.2 Animal model preparation

#### 2.2.1 Animals

Adult male Sprague-Dawley (SD) rats weighing 250–280 g used in experiment were bought from SJA Laboratory Animal Co. LTD (Hunan, China). Before experiments, rats were housed in cages (5 rats per cage) in a climate-controlled facility with 12 h light/dark cycle at constant temperature (24 ± 1 °C) and humidity (40–60%) with free access to food and water. The experimental content is consistent with national legislation and associated guidelines and the research were approved by the Animal Ethics Committee of Fujian Medical University, under laboratory animal license no. SYXK (Min) 2016-0006.

#### 2.2.2 Middle Cerebral Artery Occlusion (MCAO) model

Two hundred rats were randomly assigned into five groups based on research content: Sham group, MCAO group, MCAO + sh-EphA4 group, MCAO + EphA4-OE group and MCAO + EphA4-OE + PDTC group. Researchers were blinded to group assignment during data acquisition and related discussion.

Rat MCAO models were established as described before [10]. During the experiment, all rats inhaled 2% isoflurane for anesthesia. After incision of the skin, the common carotid artery and internal and external arteries were carefully separated. And a silicon coated nylon monofilament was inserted into the common carotid artery, and the MCA was sufficiently blocked by proper advancement. The model was perfectly constructed when the local blood flow reduced to 30% of the original value. Regional cerebral blood flow was continuously monitored with a laser Doppler flowmeter (VMS-PCB V3, Moor Factory, UK) at the MCA territory (coordinates: 5 mm lateral to the midline, 1 mm posterior to the bregma) [11]. After 1 h occlusion, the monofilament was removed to allow perfusion. Sham group rats got similar treatment as the MCAO group except for occlusion. Rectal temperature of anesthetized animal was kept at 37 ± 0.5 °C. Animals with a CBF reduction of <70% were removed from this study. Rats were also excluded if they showed surgical complications such as epilepsy. Of all the 200 animals, a total of 133 (66.5%) were available for further analysis. Among the 67 animals excluded, 43 died during or after surgery, 15 did not reach the CBF standard of ischemia and 9 have seizures after surgery.

Animals in the MCAO + sh-EphA4 group were treated with Lenti-sh-EphA4, rats in the MCAO + EphA4-OE group and MCAO + EphA4-OE + PDTC group were both delivered Lenti-EphA4-OE through intracerebroventricular (ICV) injection 30 min before MCAO. Sham group and MCAO group were given scramble shRNA control instead by the following method.

#### 2.2.3 Drug preparation and administration in vivo

In vivo, lentiviral vectors expressing nontargeting scramble controls or EphA4 shRNA as well as overexpressing EphA4 were delivered by ICV injections. Before MCAO, lentiviral vectors were diluted with transfection reagent (Entranser™, Engreen Biosystem, Ltd., China). For ICV injection, 5 μl plasmid mixtures containing 100 pmol lentiviral vectors such as Lenti control or sh-EphA4 or EphA4-OE were stereotaxically delivered. Rats were anesthetized 30 min before ischemia, and ICV injection were conducted into the right lateral ventricle (coordinates: 1.0 mm posterior, 1.4 mm lateral to the bregma, 4.0 mm dorsoventral from the surface) for 10 min. To avoid possible leakage, the syringe stayed for another 5 min before removing.

Rats in the MCAO + EphA4-OE + PDTC group got the NF-κB inhibitor PDTC (Sigma, USA) which was dissolved in PBS (pH 7.4) and given 200 mg/kg i.p. 2 h after ischemia and again 12 h later [12]. Animals in other four groups received PBS instead of PDTC at the same time point.

#### 2.2.4 Infarct volume assessment

Infarct volume was assessed by magnetic resonance imaging (MRI) method as follows: the experimental rats were anesthetized and then placed in a 7.0T MRI scanner (Bruker, Germany), and scanned with mouse specific coils. The T2WI images were processed to determine and calculate the corresponding infarct area on ImageJ 1.48v (NIH, USA) [13].

#### 2.2.5 Behavioral measurements

The neurological function was detected based on a Garcia 18-point scoring system before and on 1, 3, 7, and 14 days after ischemia as described before [14]. The scoring system include six neurological tests: (1) spontaneous activity, (2) movement of four limbs, (3) forepaw outstretching, (4) climbing, (5) body proprioception (6) response to vibrissae touch. The total score ranged from minimum 3 (most severe) to maximum18 (normal).

#### 2.2.6 Morris Water Maze (MWM) test

The hippocampal dependent spatial learning ability of animal was tested by MWM experiment, and appropriate improvements were also made according to the requirements of this study [15]. The water maze (120 cm in diameter) applied in the experiment was divided into 4 quadrants. A platform, about 9 cm in diameter, was set at the center of one quadrant, and hided 1 cm under water. During the pre-training, the rats were placed into the maze to find hidden platform, in the experiment process, their escape latency was recorded. If the rat did not find the target within 1 min, in this case, it would be redirected to the platform and kept stayed on it for 20 s [15]. Animals were trained to search hidden platform from 4 starting quadrants in each training. All rats had received pre-training for 4 days before MCAO. All animals underwent 6 MWM trials on postoperative day 8–10. The escape latency of rat was recorded and averaged to get one trial score.

#### 2.2.7 Immunofluorescent staining

The cellular distributions of proteins were detected based on immunofluorescent staining technology [5]. For post-fixation, the brain sections or cell cultures were immersed into 100% cold methanol for half an hour. After blocking nonspecific binding site with 10% BSA/PBS, samples were incubated with primary antibodies (anti-EphA4, 1:100, Santa Cruz Biotechnology, Cat. No. sc-135897, RRID: AB_2099356; anti-MAP-2, 1:50, Cell Signaling Technology, Cat. No. 4542, RRID: AB_10693782; anti-NeuN, 1:200, Abcam, Cat. No. ab177487, RRID: AB_2532109) at 4 °C overnight. The samples were next incubated with appropriate secondary antibodies (CY3-conjugated donkey anti-mouse IgG antibody, 1:400, Jackson ImmunoResearch Laboratories, Cat. No. 715-165-150, RRID: AB_2340813; FITC-conjugated goat anti-rabbit IgG antibody, Cat. No. 111-095-003, RRID: AB_2337972) at 25 °C for 1 h. Then brain sections or cell cultures were washed, dried and mounted with a coverslip. At last, the results of immunofluorescent staining were observed with Olympus BX51 fluorescent microscope (Olympus, Japan).

#### 2.2.8 TUNEL staining

The neuronal cell apoptosis were detected by Apoptag Red Kit (Roche, USA) based on TUNEL staining method. In the process of this experiment, brain sections were post-fixed with the ethanol and acetic acid (2:1) mixture at −20 °C for 5 min, then incubated with TdT enzyme at 37 °C for 1 h. After reaction terminated, bran sections were incubated with antidigoxigenin antibody at 25 °C for half an hour. Brain sections were further stained with DAPI (1 μg/ml, Sigma, USA). At last, sections were mounted with a coverslip. The TUNEL-positive cells in peri-ischemic regions were calculated and averaged from 4 randomly chosen view-fields under Olympus BX51 fluorescent microscope (Olympus, Japan).

#### 2.2.9 Primary neuronal culture

A total of 72 mixed-gender P0 or P1 SD rats used for primary neuronal culture were purchased from Experimental Animal Center of Fujian Medical University. Primary neurons were got from P0 or P1 SD rats as described before with mirror modifications [16]. Both cerebral cortices and hippocampi were carefully isolated and then dissociated by 0.125% trypsin (Gibco, USA). Dissociated cells were seeded onto poly-l-lysine (Sigma, USA) coated 24-well plate containing neurobasal medium with additional 2% B27 (Gibco, USA) with the cell density of 10^5^ cells/well. MAP-2 (2 μg/ml, Abcam. ab11267, RRID: AB_297885) and DAPI (1 g/ml, Sigma) double-labelling staining was carried out to detect the neuron purity. Primary neuron cultures with 95% purity or more can be used in the subsequent experiments.

#### 2.2.10 Primary microglial culture

A total of 48 mixed-gender P0 or P1 SD rats used for primary microglial culture were purchased from Experimental Animal Center of Fujian Medical University. Microglia were separated from mixed glial cultures as described before [17]. In this experimental process, cerebral cortices and hippocampi of P0 or P1 SD rat were carefully isolated, and then dissociated by 0.25% trypsin. The cells were seeded in culture flasks containing DMEM/F12 (1:1) with additional 10% FBS. After 10 days of culture, flasks were shaken (37 °C, 200 rpm, 1.5h) to collect supernatant containing microglia. Supernatant cells were then plated onto glass coverslips with the cell density of 5 × 10^6^ cells/cm^2^. After 30 min, microglia were separated as tightly adherent cells. Iba-1 (1:200, Abcam, Cat. No. ab178847, RRID: AB_2832244) and DAPI double-labelling staining was carried out to test the microglia purity. Primary microglia cultures with 95% purity or more were applicable for subsequent experiments.

#### 2.2.11 Neuron-microglia co-culture system and oxygen glucose deprivation (OGD) treatment

In this experiment, purified microglial cells were seeded into primary neurons at DIV 5 with the cell density of 5 × 10^4^ cells/well in a 24-well plate. Three hours later, co-cultures suffered OGD to mimic ischemia in vitro. Briefly, culture medium was replaced by serum and glucose-free DMEM, and co-cultures were placed in an anaerobic chamber (Sigma, USA) filled with 93% N_2_, 5% CO_2_ and 2% O_2_ for 1 h. Then co-cultures returned back into a normoxic chamber and cultured in normal culture medium. The cells were collected after 24 h to provide support for subsequent experiments.

#### 2.2.12 Drug preparation and administration in vitro

Primary neurons in the OGD + Lenti Control group or OGD + sh-EphA4 group or OGD + EphA4-OE group or OGD + EphA4-OE + PDTC group were transfected with specific lentiviral vectors before co-culture. In the OGD + EphA4-OE + PDTC group, PDTC was premixed with culture medium at a concentration of 50 μM 3 h before the OGD model was induced.

### 2.3 Flow cytometry analysis for OGD-induced apoptosis

Flow Cytometry method was applied to measure the apoptotic cells with application of Annexin V-Fluorescein Isothiocyanate (FITC) Early Apoptosis Detection Kit (Cell Signaling Technology, USA) following instruction provided. Briefly, cells were trypsinized and then resuspended. Cell suspensions were incubated with Annexin V-FITC and propidium iodide (PI) for 10 min at room temperature (RT) and avoid light. Samples should be detected by flow cytometry (Beckman FC 500, USA) timely. Necrotic cells (Quadrant UL), late apoptotic cells (Quadrant UR) and early apoptotic cells (Quadrant LR) and were counted separately.

#### 2.3.1 EdU staining

To indicate cell proliferation, neuron-microglia co-cultures were stained EdU (5-ethyny1–20-deoxyuridine) with a Click‐iT EdU Imaging Kit (Invitrogen, USA) 24h after OGD treatment according to operating instruction.

#### 2.3.2 Quantitative RT-PCR

RNeasy Mini kit (Qiagen, China) were applied to extract the total RNA. A First Strand cDNA Synthesis Kit (Thermo Scientific, USA) was used for reverse transcription reaction. The primers were purchased from Sangon Biotech (Shanghai, China). PCR was conducted on the ABI Prism7500 sequence detection system (Applied Biosystems, USA) at 95 °C for 1 min, then 40 cycles at 95 °C for 20 s, at 56 °C for 20 s, and at 72 °C for 30 s. The SYBR Green PCR Master Mix (Takara, Japan) was used to detect the PCR products. Data were processed by 2-ΔΔ cycle threshold (CT) method [18]. GAPDH was set as an internal control.

#### 2.3.3 Western blot (WB) analysis

To investigate changes in protein expression, WB analysis was applied. Briefly, total protein was extracted from harvested cells using 1% CHAPS (Sigma, USA) lysis buffer containing PMSF (Sigma, USA) and protease inhibitor cocktail (Roche, Germany). During this experiment, the cell lysates were centrifuged at 12000 g for 10 min at 4 °C, after that, the obtained supernatant was fully collected to provide support for subsequent experiments. The bicinchoninic acid (BCA) kit (Boster, China) was applied for protein quantitation. To detect NF-κB p65 nuclear translocation, the Nuclear Extraction Kit (Beyotime, China) was applied to extract nuclear proteins. After proteins with different molecular weight were separated by gel electrophoresis, the target protein was transferred onto the nitrocellulose membrane (0.45 μm, Millipore, USA) through a wet transfer system (Bio-Rad, USA). After blocking the non-specific binding sites for 1.5 h, the membranes were washed, and then incubated with the corresponding primary antibodies (anti-β-tubulin, 1:500, Abcam, Cat. No. ab6046, RRID: AB_2210370; anti-EphA4, 1:200, Santa Cruz Biotechnology, Cat. No. sc-135897, RRID: AB_2099356; anti-iNOS, 1:1000, Thermo Fisher Scientific, Cat. No. PA5-17106, RRID: AB_10981485; anti-Arg1, 1:1000, Cell Signaling Technology, Cat. 93668, RRID: AB_2800207; anti–NF–κB p65, 1:1000, Cell Signaling Technology, Cat. No.6959, RRID: AB_10891780; anti-Phospho–NF–κB p65, 1:1000, Cell Signaling Technology, Cat. No.3031, RRID: AB_330559; anti-IκBα, 1:1000, Cell Signaling Technology, Cat. No.4812, RRID: AB_10694416; anti-Phospho-IκBα, 1:1000, Cell Signaling Technology, Cat. No.9246, RRID: AB_2267145; anti-Histone H3, 1:1000, Abcam, Cat. No. ab1791, RRID: AB_302613) at 4 °C for 12 h. Then the membranes were sufficiently washed, and then incubated with the corresponding secondary antibodies (anti-mouse horseradish peroxidase-conjugated, 1:5000, Abcam, Cat. No. ab205719, RRID: AB_2755049; anti-rabbit horseradish peroxidase-conjugated, 1:5000, Santa Cruz Biotechnology, Cat. No. sc-2357, RRID: AB_628497). Finally, an ECL kit (Thermo Fisher Scientific, USA) was used to detect membranes. The digital images of results were processed by Image J. Either β-tubulin or Histone H3 acted as an internal control.

#### 2.3.4 Enzyme-Linked Immunosorbent Assay (ELISA)

The cytokine levels of IL-1β, IL-4, IL-6, IL-10, TNF-α and TGF-β in the co-cultures were detected with ELISA kit (Invitrogen, USA). Basing on the protocol, the standard reference curve was drawn according to experiment result for estimating cytokine levels in samples.

#### 2.3.5 Electrophoretic Mobility Shift Assay (EMSA)

Nuclear extracts were obtained from neuron-microglia co-cultures using Nuclear Extraction Kit (Beyotime, P0028-3). In this experiment, 5 × 10^6^ cells were suspended and lysed with 10% NP-40. After centrifugation, the supernatant extracts were collected and then resuspended in nuclear extraction on ice. After full mixing for 30 min, the supernatant nuclear extracts were separated by centrifugation.

The EMSA Kit (Thermo Fisher, USA) was used for studying DNA-protein interactions. Briefly, 3 μg nuclear extract was incubated with biotinylated probe for 30 min at RT. The complex was separated and transferred to a nylon membrane, after that the transferred DNA was crosslinked on the membrane, and the labeled DNA was detected and analyzed, and the results were recorded.

### 2.4 Statistical analysis

The data collected were statistically analyzed using SPSS 19.0 software. The Kolmogorov-Smirnov test was used to determine data normality. Most data were normally distributed except for data of neurological function score. For normally distributed data, the results were presented as the mean ± SD. One-way analysis of variance (ANOVA) followed by LSD's post hoc test was carried out to determine whether there were statistical differences between groups. For data not normally distributed, the results were presented as the median ± interquartile range, and the nonparametric Kruskal-Wallis followed by Bonferroni correction was used to compare statistical differences between groups. The judgment standard was set as *P* < 0.05.

## 3 Results

### 3.1 Cellular distribution and change pattern of EphA4 after transient focal ischemia

**Fig. 1:**
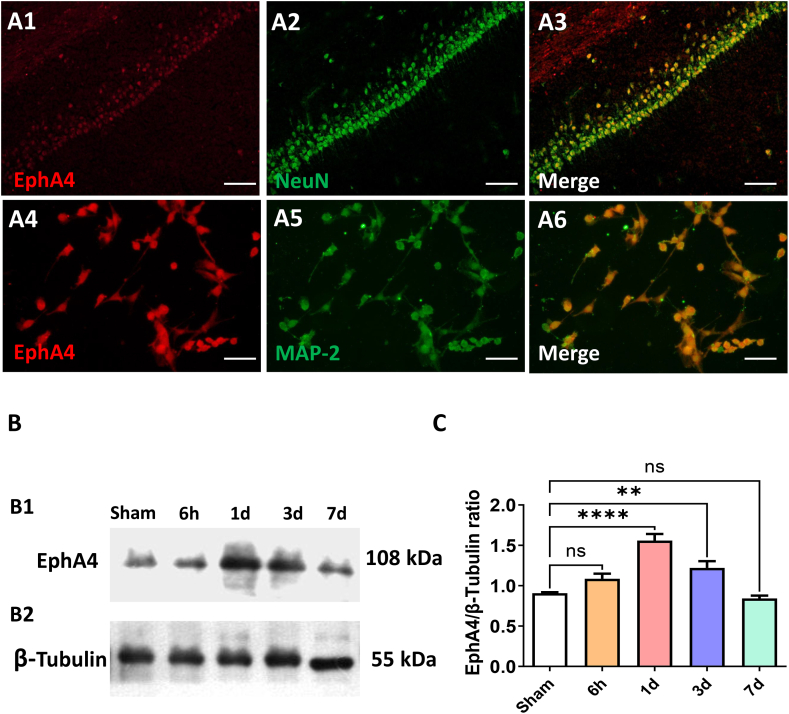
Cellular Distribution and Change Pattern of EphA4 in Rat Brain. (A1-A3) Double-label immunofluorescent staining of EphA4 (red) and NeuN (green) in CA1 region of hippocampus. Scale bar = 50 μm (A4-A6) EphA4 expression on primary neurons identified by double immunofluorescent staining with EphA4 (red) and MAP-2 (green). Scale bar = 50 μm (B1–B2) The change pattern of EphA4 after transient focal ischemia was detected by Western Blot. (C) Quantifications of EphA4 protein levels. The results were represented as the mean ± SD, and the differences between groups were compared with one-way analysis of variance (ANOVA) followed by LSD's post hoc test (n = 5, **P < 0.01, ****P < 0.0001 vs Sham group). (For interpretation of the references to colour in this figure legend, the reader is referred to the Web version of this article.)

Cellular localization of the EphA4 was confirmed by immunofluorescent double labeled staining. The relevant result was shown in Fig. 1A, this kind of receptor mainly located in the NeuN/MAP-2-positive neurons both in vivo (Fig. 1 A1-A3) and in vitro (Fig. 1 A4-A6).

The temporal pattern of EphA4 receptor protein expression in peri-ischemic regions of rats after transient focal ischemia was verified based on WB method. After transient MCAO, the EphA4 protein level started to upregulate at 6 h and peaked on day 1, then gradually downregulated on day 3 and day 7. The expression of EphA4 protein was significantly higher than that of sham group on day 1 and day 3 after ischemia (*P* < 0.01) (F = 21.74) (Fig. 1 B1–B2, C).

#### 3.1.1 EphA4-OE lentiviral vectors construction

**Fig. 2:**
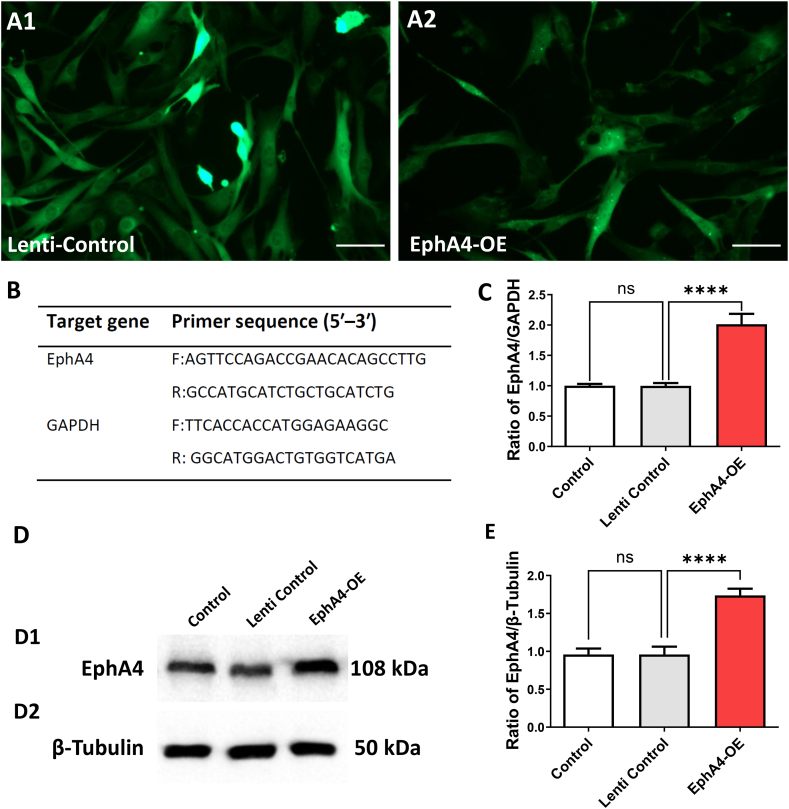
EphA4 Overexpression Lentiviral Vectors Construction. (A1-A2) The GFP (green) staining in primary neuron cultures which confirmed that lentiviral vectors that expressing nontargeting controls or overexpressing EphA4 were transfected into primary neurons. Scale bar = 50 μm. (B) Primers for detecting target gene. (C) The EphA4-specific overexpression lentiviral vectors increased EphA4 mRNA level, as confirmed by RT- PCR. EphA4 mRNA level was quantified and normalized to GAPDH. (D1-D2) The EphA4 protein level was tested by Western Blot method. (E) EphA4 protein expression was quantified and normalized. The results were represented as the mean ± SD, and the differences between groups were compared with ANOVA followed by LSD's post hoc test (n = 5, ****P < 0.0001 vs Lenti Control group). (For interpretation of the references to colour in this figure legend, the reader is referred to the Web version of this article.)

As shown in Fig. 2A, lentiviral vectors expressing nontargeting controls (Fig. 2 A1) or overexpressing EphA4 (Fig. 2 A2) were transfected into primary neurons and indicated by GFP. Primers for detecting EphA4 gene were shown in Fig. 2B. The EphA4 mRNA and protein expression levels in primary neurons were evaluated by RT-qPCR and WB method. There were no significant differences between Control and Lenti Control group in EphA4 mRNA and protein expression levels. The EphA4 mRNA level in EphA4-OE group showed a nearly 2-fold increase than the Lenti Control group (*P* < 0.01) (F = 304.69) (Fig. 2C). Similarly, the EphA4 protein level in the EphA4-OE group was obviously higher than the Lenti Control group (*P* < 0.01) (F = 229.52) (Fig. 2 D1-D2, E).

#### 3.1.2 EphA4 overexpression aggravated neuronal damage and worsened neurobehavioral function after ischemia

**Fig. 3:**
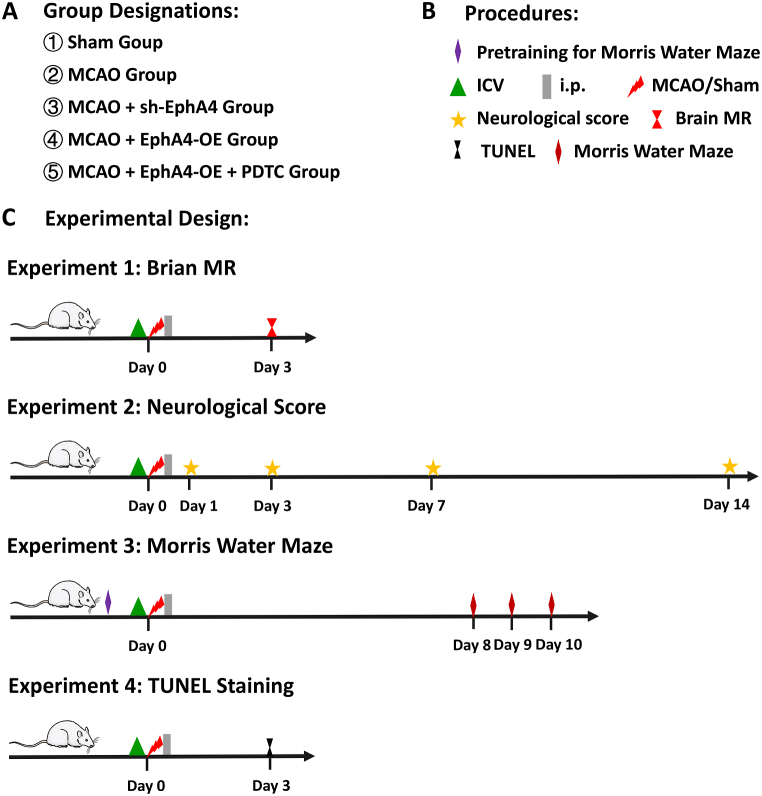
Schematic diagram of experimental procedure. (A) Animals were randomly assigned into five groups: Sham group, MCAO group, MCAO + sh-EphA4 group, MCAO + EphA4-OE group and MCAO + EphA4-OE + PDTC group. (B) Representative mark of procedures carried out during animal experiments. (C) Separate series of 4 main animal experiments including infarct volume assessment with Brain MRI, neurological function scoring, Morris Water Maze test and TUNEL staining.

**Fig. 4:**
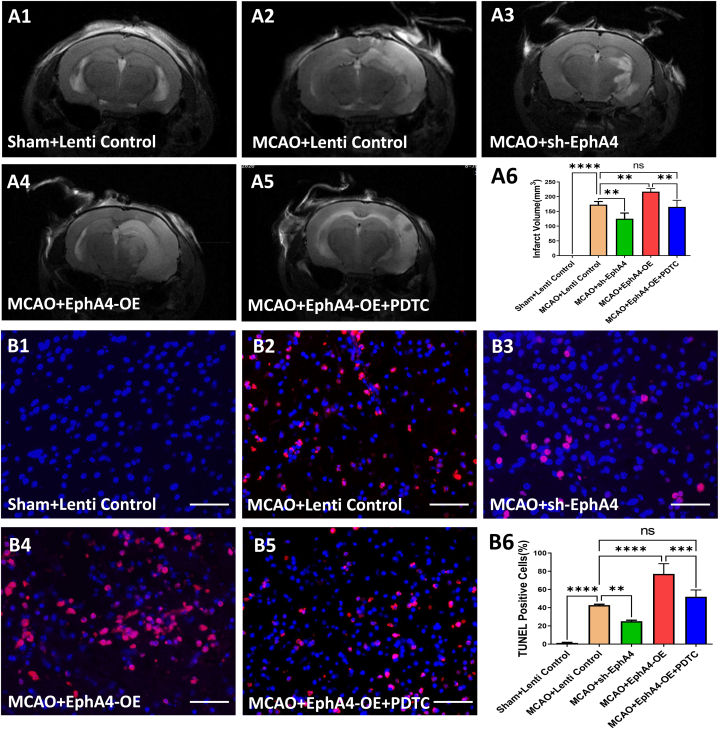
EphA4 Overexpression Aggravated Brain Damage and Neuronal Apoptosis after Ischemia. (A1-A5) Representative MRI images showing infarcted areas after MCAO. (A6) Infarct volume evaluated at day 3 after MCAO. (B1–B5) TUNEL (+) staining (red) plus DAPI (blue) to label apoptotic cells in the boundary zone of ischemic area. Scale Bar = 50 μm (B6) Counting of TUNEL-positive cells in peri-ischemic regions of MCAO rats on postoperative day 3. The results were represented as the mean ± SD, and the differences between groups were compared with ANOVA followed by LSD's post hoc test (n = 5, **P < 0.01, ***P < 0.001, ****P < 0.0001, ns: no significance). (For interpretation of the references to colour in this figure legend, the reader is referred to the Web version of this article.)

To study the EphA4/ephrin bidirectional signaling, we manipulated neuronal EphA4 with either sh-EphA4 or EphA4-OE lentiviral vectors. A schematic diagram of experimental procedure with a time axis for each group has been included in Fig. 3 (A-C). We found that sh-EphA4 application significantly decreased infarction size (*P* < 0.01) (F = 281.45) (Fig. 4 A1, A2, A3, A6) after ischemia as well as ischemia-induced neuronal apoptosis evidenced by TUNEL staining in the peri-ischemic areas than the MCAO + Lenti Control group (*P* < 0.01) (F = 187.89) (Fig. 4 B1, B2, B3, B6). On the contrary, EphA4-OE significantly increased infarction size together with ischemia-induced neuronal apoptosis (*P* < 0.01) (Fig. 4 A1, A2, A4, A6, B1, B2, B4, B6). Both the infarction area and ischemia-induced neuronal apoptosis deteriorated by EphA4-OE could be redeemed by NF-κB inhibitor PDTC (*P* < 0.01), and there were no significant differences between MCAO + EphA4-OE + PDTC group and MCAO + Lenti Control group in infarction size as well as ischemia-induced neuronal apoptosis (Fig. 4 A2, A4, A5, A6, B2, B4, B5, B6).

**Fig. 5:**
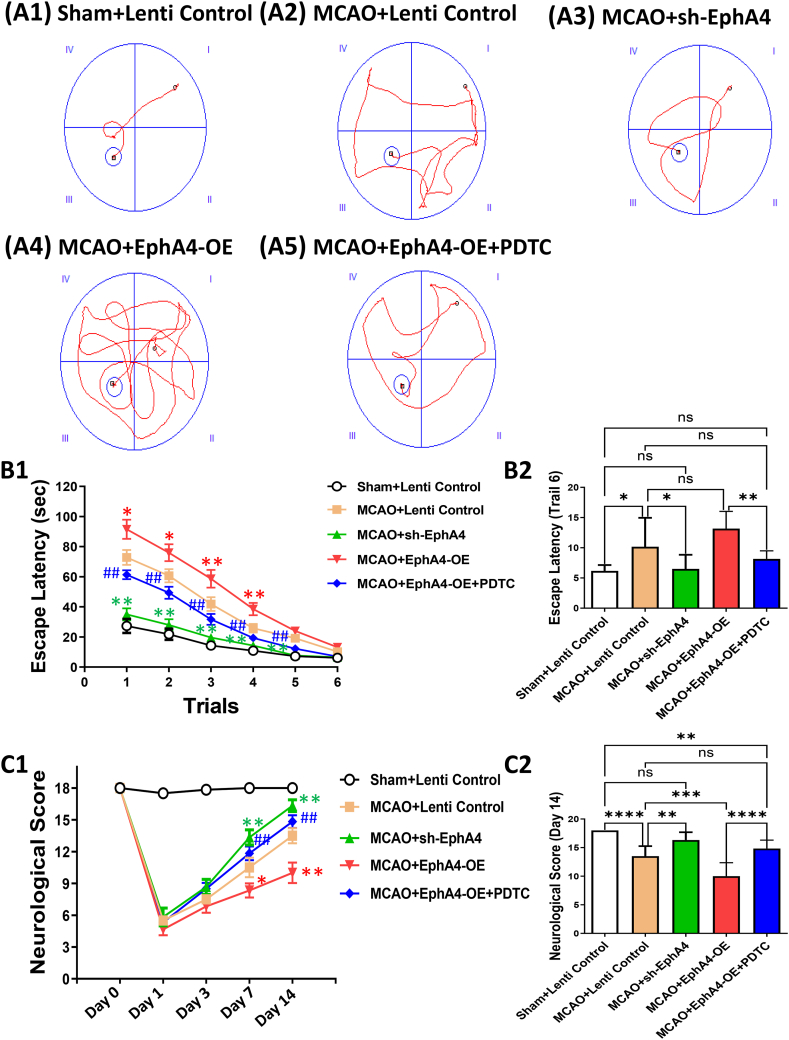
EphA4 Overexpression Worsened Neurobehavioral Function after Ischemia. (A1-A5) Representative swimming traces in MWM from different groups. (B1–B2) Escape latency, which indicated hippocampus-dependent spatial memory, was measured. The results were represented as the mean ± SD, and the differences between groups were compared with ANOVA followed by LSD's post hoc test (n = 6, *P < 0.05, **P < 0.01, ***P < 0.001, ****P < 0.0001, ns: no significance). (C1–C2) Neurological deficit was scored. The results were represented as the mean ± SD or median ± interquartile range, and ANOVA followed by LSD's post hoc test or nonparametric Kruskal-Wallis followed by Bonferroni correction was used to compare differences between groups (n = 6, *P < 0.05, **P < 0.01, ***P < 0.001, ****P < 0.0001, ns: no significance).

This paper further discussed the influence of EphA4 silencing and EphA4 overexpression on neurological function. Animals all showed obvious motor function impairment after ischemia, but experienced partial recovery on 3–14 d. Ischemia induced neurobehavioral deficits was significantly worsened by EphA4-OE on days 7 and days 14 (*P* < 0.05) comparing with the control-treated groups, which could also be saved by PDTC (*P* < 0.01). EphA4 shRNA treated group showed quite the opposite effect of the EphA4-OE group on post-ischemia day 7–14 (*P* < 0.01) (Fig. 5 C1). It was worth mentioning that no significant differences were observed between Sham + Lenti Control group and MCAO + sh-EphA4 group as well as MCAO + Lenti Control group and MCAO + EphA4-OE + PDTC group on days 14 (Fig. 5 C2) (F = 105.13 for Day 1, 72.84 for Day 3; χ2 = 22.64 for Day 7; 23.91 for Day 14).

MWM test further demonstrated that rats in the MCAO + sh-EphA4 group performed better to locate the platform than in the MCAO + Lenti Control group during the whole 6 trials (*P* < 0.01). According to the experimental results, it was also found that, rats receiving EphA4-OE before ischemia showed significantly prolonged escape latency during the first 4 trials, comparing to rats receiving control therapy (*P* < 0.05), suggesting that EphA4 overexpression aggravated the ischemia induced injury of hippocampal relative spatial memory. Application of NF-κB inhibitor PDTC ameliorated memory impairment induced by EphA4-OE (*P* < 0.01), revealing that NF-κB is crucial in signaling pathways downstream of EphA4/ephrin (Fig. 5 A1-A5, B1). When it came to the last trail, animals in both MCAO + sh-EphA4 group and MCAO + EphA4-OE + PDTC group performed as well as animals in the Sham + Lenti Control group (Fig. 5 B2) (F = 31.75 for Trail 1, 26.48 for Trail 2, 19.03 for Trail 3, 14.7 for Trail 4, 13.65 for Trail 5, 6.86 for Trail 6).

#### 3.1.3 EphA4 overexpression increased apoptosis and microglia proliferation induced by OGD

**Fig. 6:**
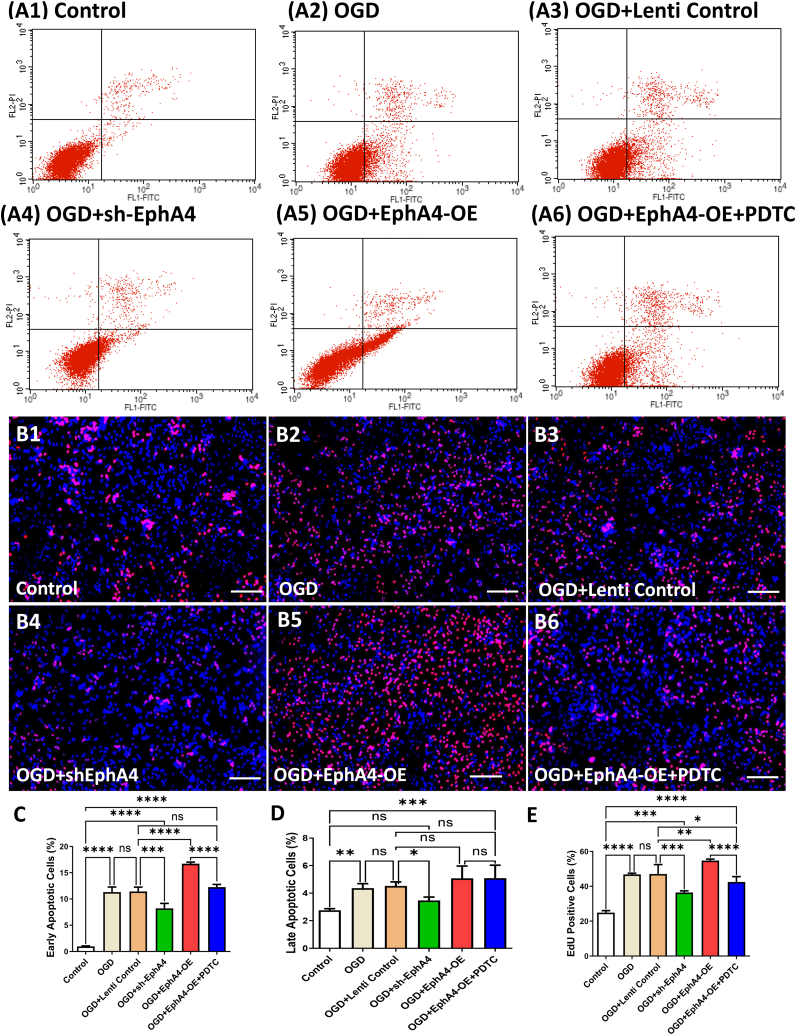
EphA4 Overexpression Increased Apoptosis and Microglia Proliferation Induced by OGD. (A1-A6) Representative data of apoptosis detected by flow cytometry. (B1–B6) Microglia proliferation was tested via EdU (red) staining and DAPI staining (blue). Scale bar = 100 μm. (C–D) Summary of the percentage of apoptotic cells in each group. (E) Counting of EdU-positive microglia cells in co-cultures. The results were represented as the mean ± SD, and the differences between groups were compared with ANOVA followed by LSD's post hoc test (n = 5, *P < 0.05, **P < 0.01, ***P < 0.001, ****P < 0.0001, ns: no significance). (For interpretation of the references to colour in this figure legend, the reader is referred to the Web version of this article.)

To further study the potential mechanisms of EphA4/ephrin signaling in ischemia in vitro, we applied sh-EphA4 and EphA4-OE to manipulate EphA4. As we saw in Fig. 6 A1-A6, EphA4 shRNA exhibited a protect effect on OGD-induced early and late apoptosis (*P* < 0.05) (Fig. 6 C, D). On the contrary, EphA4-OE significantly increased early apoptosis caused by OGD (*P* < 0.01) (F = 395.64) (Fig. 6 C), but the late apoptosis between EphA4-OE group and OGD + Lenti Control group showed no significance as demonstrated by flow cytometry analysis (F = 21.74) (Fig. 6 D). EdU staining showed that EphA4-OE application also aggravated the OGD-induced microglia proliferation (*P* < 0.01) which also could be improved by sh-EphA4 (*P* < 0.01) (Fig. 6 B1–B6). We further found that neuronal apoptosis (*P* < 0.01) as well as microglia proliferation (*P* < 0.01) increased by EphA4-OE could be reversed by NF-κB pathway inhibitor PDTC (Fig. 6 C, E). Comparing with non-treated control group, OGD + sh-EphA4 group showed a significantly elevated early apoptotic rate (*P* < 0.01) (Fig. 6 C) but a similar late apoptotic rate (Fig. 6 D). In the OGD + EphA4-OE + PDTC group, both early apoptosis and late apoptosis were more significant than in the non-treated control group (*P* < 0.01) (Fig. 6 C, D). The EdU positive rates of OGD + sh-EphA4 group and OGD + EphA4-OE + PDTC group were significantly higher than that of non-treated control group (*P* < 0 0.01) (F = 102.35) (Fig. 6 E).

#### 3.1.4 Effect of EphA4 overexpression on microglia activation

**Fig. 7:**
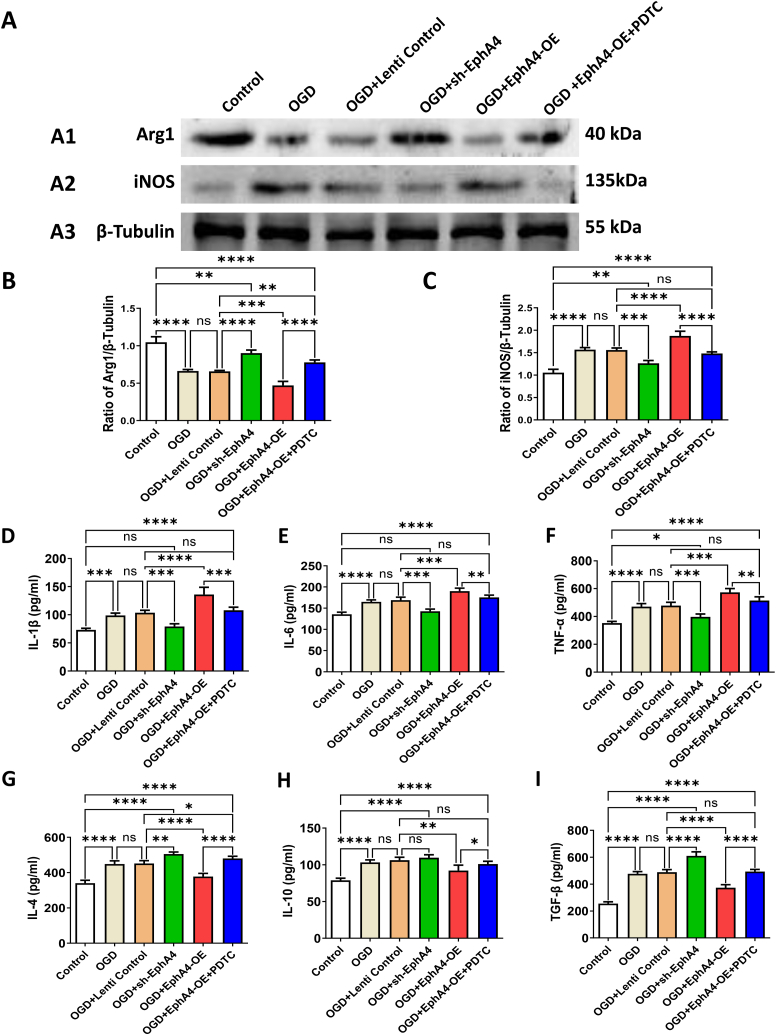
Effects of Neuronal EphA4 Manipulation on The Polarization of Microglia. (A1-A3) Western Blot indicated protein level changes in Arg1 and iNOS. (B and C) Quantification of the protein levels of the Arg1 (B) and iNOS (C). Protein level was quantified and normalized to β-Tubulin. (D–I) Cytokine levels of IL-1β (D), IL-6 (E), TNF-α (F), IL-4 (G), IL-10 (H) and TGF-β (I) were detected by ELISA kit. The results were represented as the mean ± SD, and the differences between groups were compared with ANOVA followed by LSD's post hoc test (n = 5, *P < 0.05, **P < 0.01, ***P < 0.001, ****P < 0.0001, ns: no significance).

As the increased cell number could not give the full picture of microglia activation states, the role of EphA4 overexpression on microglia activation was investigated by detecting iNOS and Arg1, which are the widely accepted subtype markers distinguishing microglial M1 and M2 phenotypes separately [19,20]. Compared with the non-treated control group, both iNOS and Arg1 expressions changed significantly after OGD in all groups (*P* < 0.01). With EphA4-OE application before OGD, Arg1down-regulation (*P* < 0.01) (F = 191.68) and iNOS up-regulation (*P* < 0.01) (F = 139.92) were enhanced (Fig. 7 A1-A3, B, C). The above results indicated that neuronal EphA4 overexpression promoted microglia classically activating (M1) rather than alternative activating (M2).

Cytokine level changes were further detected by ELISA to study the microglial secretory function. The EphA4-OE treatment further increased the secretion level of IL-1β, IL-6 and TNF-α induced by OGD (*P* < 0.01) (F = 102.07 for IL-1β, 95.41 for IL-6, 103.17 for TNF-α). Cytokines secretion level of IL-4, IL-10 and TGF-β also enhanced obviously after OGD. With EphA4-OE treatment before OGD, the expressions of IL-4, IL-10 and TGF-β were all lower than the OGD + Lenti Control treated group (*P* < 0.01) (F = 130.44 for IL-4, 48.11 for IL-10, 291.92 for TGF-β). Results above revealed that EphA4 overexpression affected microglial secretory function, and promoted inflammatory cytokines secretion.

Comparing with the EphA4-OE group, sh-EphA4 group show the exactly the opposite effect on phenotypic transformation of microglia (*P* < 0.01) as well as secretory function of microglia (*P* < 0.01). After sh-EphA4 treatment, cytokine secretion of IL-1β and IL-6 in the OGD + sh-EphA4 group returned to the similar level as the non-treated control group. We also found NF-κB pathway inhibitor PDTC could reverse the effect EphA4-OE application exhibited on microglial phenotype switch and secretory function (*P* < 0.01) (Fig. 7 D-I, Table 1).

**Table 1:** Cytokine alterations in different treatment groups (pg/ml).

	Control	OGD	OGD + Lenti Control	OGD + sh-EphA4	OGD + EphA4-OE	OGD + EphA4-OE + PDTC
IL-1β	73.00 ± 2.22	98.72 ± 3.13	103.66 ± 3.38	79.09 ± 3.87	135.96 ± 9.39	107.85 ± 4.42
IL-6	135.61 ± 4.24	164.95 ± 3.77	168.77 ± 5.92	142.53 ± 4.27	190.15 ± 5.28	175.35 ± 4.32
TNF-α	352.25 ± 10.42	470.59 ± 16.94	477.73 ± 19.18	397.19 ± 15.08	572.72 ± 20.53	514.38 ± 20.56
IL-4	340.45 ± 12.45	448.78 ± 13.9	451.51 ± 13.26	505.23 ± 9.32	377.76 ± 13.83	480.09 ± 10.06
IL-10	78.98 ± 2.56	103.32 ± 2.82	106.29 ± 3.72	109.63 ± 3.00	92.22 ± 5.72	101.01 ± 3.10
TGF-β	255.78 ± 10.42	477.11 ± 13.39	489.17 ± 14.65	610.56 ± 23.74	373.43 ± 17.15	493.43 ± 12.29

Data were presented as mean ± standard deviation.

#### 3.1.5 EphA4 overexpression shifting microglia M1-polarization via NF-κB signaling

Previous research had confirmed the association between EphA/ephrin signaling and downstream NF-κB. NF-κB is a kind of very important nuclear transcription factor, contributing to neuroinflammation through regulating microglia [21,22]. In the further research, we explored if neuronal EphA4 manipulation switched the microglia polarization via downstream NF-κB signaling.

**Fig. 8:**
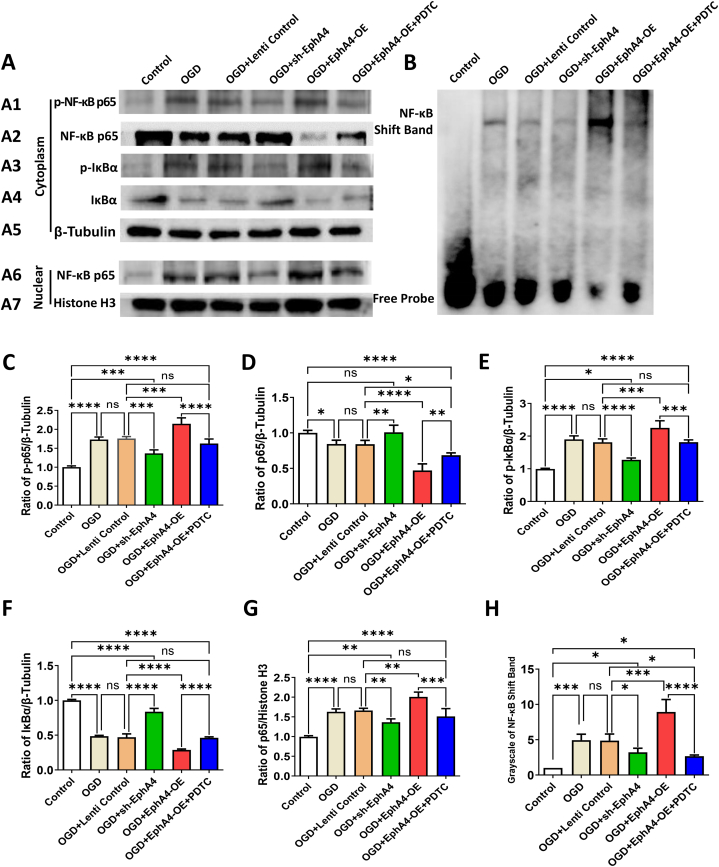
EphA4 Manipulation Regulated the Microglia Polarization via NF-κB Signaling. (A1-A7) Representative Western blot images showed the protein levels of p–NF–κB p65, total NF-κB p65, p-IκBα, total IκBα and nuclear NF-κB p65. The β-Tubulin and histone H3 acted as loading control for cytoplasmic and nuclear proteins respectively. (B, H) EMSA with a DIG-labeled NF-κB probe was performed to explore the DNA binding activity of NF-κB. (C–G) Quantification of the protein levels of the cytoplasmic p–NF–κB p65 (C), cytoplasmic total NF-κB P65 (D), cytoplasmic p-IκBα (E), cytoplasmic total IκBα (F) and nuclear NF-κB p65 (G). The results were represented as the mean ± SD, and the differences between groups were compared with ANOVA followed by LSD's post hoc test (n = 5, *P < 0.05, **P < 0.01, ***P < 0.001, ****P < 0.0001, ns: no significance).

After OGD, NF-κB pathways activated as indicated by a significant increase of p–NF–κB p65 and a slight decrease of total NF-κB p65. Comparing with the non-treated control group, NF-κB pathway activations enhanced significantly after OGD in all groups (*P* < 0.01). Upon EphA4-OE treatment, NF-κB pathways activation was more remarkable than the OGD + Lenti Control group (*P* < 0.01) (F = 151.05 for p-p65, 69.3 for p65) (Fig. 8 A1, A2, A5, C, D). The existing experimental results show that IκB-α, the inhibitory subunit of NF-κB complex, is very important in NF-κB nuclear translocation. In comparison to non-treated control group, we found that IκB-α phosphorylation and degradation were remarkably promoted after OGD in all groups. EphA4-OE application further increased the IκB-α phosphorylation and degradation on basis of OGD (*P* < 0.01) (F = 137.93 for p-IκB-α, 608.41 for IκB-α) (Fig. 8 A3, A4, A5, E, F). Comparing with the EphA4-OE group, sh-EphA4 group had the exactly opposite effect on NF-κB signaling activation (*P* < 0.05) (Fig. 8 A, C–F). And the EphA4-OE + PDTC (*P* < 0.01) treatment groups showed reversal of the EphA4-OE group trend (Fig. 8 A, C–F). The same trend as p–NF–κB p65 level was observed on NF-κB p65 translocation indicated (*P* < 0.01) (Fig. 8 A6, A7, G).

Furthermore, EMSA with a DIG-labeled NF-κB probe was applied to study the influence of EphA4 overexpression on the nuclear DNA binding activity of NF-κB. Our results showed that NF-κB DNA binding ability strengthened significantly after OGD in all groups (*P* < 0.01).

A remarkable increased DNA binding ability in EphA4-OE group than in OGD + Lenti Control group (*P* < 0.01). In contrast, treatment with EphA4 shRNA (*P* < 0.05) or PDTC (*P* < 0.01) decreased NF-κB DNA binding ability (F = 54.77) (Fig. 8 B, H).

## 4 Discussion

EphA4 receptor, the richest Eph receptor in CNS, is mostly expressed in neurons [4,[23], [24], [25]]. Our previous studies showed that neuronal EphA4 expression was notably up-regulated after ischemia and affected post-ischemia brain damage through controlling excitatory toxicity of glutamate as well as microglia-mediated inflammatory injury [5,6]. However, it is still not very clear for the role of neuronal EphA4 in microglial function.

This research identified that neuronal EphA4 overexpression deteriorate inflammatory injury after ischemia through promote microglia M1-polarization. The main research concern is the underlying mechanism by which EphA4 promotes microglia M1 polarization. Recent study has implied that EphA4 may regulate the expression of pro-inflammatory gene through NF-κB pathways in TBI model [8]. Consistent with previous findings, we found that EphA4 overexpression markedly promoted microglia M1 polarization as well as NF-κB pathway activation. And inflammatory factors, such as TNF-α, produced by activated M1 type microglia can form a vicious cycle of neuroinflammation and exacerbate ischemia-induced inflammatory injury. Conversely, EphA4 knockdown promoted microglial M2 polarization and inhibited NF-κB pathway after ischemia. This could reduce the expression of related inflammatory factor genes, have a good effect on inhibiting inflammatory response, and on this basis, reduce the adverse effects caused by ischemia, and play a protective role in brain tissue.

The NF-κB is the indispensable transcription factor related to the multiple aspects of innate and adaptive immune response. At rest, cytoplasmic NF-κB is binds to IκBα, an inhibitor protein in the IκB family, forming tight complexes. Once being activated by various extracellular and intracellular stimulation, IκB kinase (IKK) subsequently triggers degradation of IκBα. The NF-κB p65 subunit liberates and translocates to nucleus, where NF-κB can regulate gene transcription [26,27]. The activation is preemptive, and the possibility of microglial M1 phenotype conversion will be significantly increased [[28], [29], [30]]. In this study, we found that the ability of EphA4 to promote microglia polarized to M1 phenotype was abrogated by blocking NF-κB implying that microglia polarization to M1 phenotype appears through activation of the NF-κB pathway.

It can be inferred that neuronal EphA4 exerts its effect on brain damage via other means, for instance, activation of EphA4 forward signaling. Though EphA4/ephrin bidirectional signaling could simultaneously activate Eph receptor-expressing as well as ephrin ligand-expressing cells. Previous studies have demonstrated that EphA4 forward signaling inhibition mainly exert its effects on neuronal protection through reducing neuronal apoptosis [31,32] and improving synaptic plasticity [33,34]. Other than that, NF-κB can exert diverse functions in many different kinds of cell types in central and peripheral nervous system. However, previous study found that there is hardly any NF-κB expression in neurons. Meanwhile, induced NF-κB activity was quite lower in neurons than in other cell types [35]. Furthermore, NF-κB, a major regulator of inflammatory response, has been proved to show cell-type specificity function and mainly function through manipulating synaptic plasticity in neurons [21,36,37]. Though its specific mechanism has not been fully elucidated yet, which needs to be further studied to provide support for the optimization of relevant treatment schemes. It is conceivable that changes in microglia polarization and neuroinflammation is the potential mechanism might explain the effect neuronal EphA4 upregulation bring on brain damage after ischemia. There are two major limitations in this study. First, we mainly focused on the post-ischemia inflammatory injury regulated by EphA4/ephrin signaling between neuron and microglia, but we could not exclude other possibilities, such as neuronal EphA4 might simultaneously act through astrocytic ephrin. Second, we have not determined the exact microglial ephrin ligand through which neuronal EphA4 take effect as well as the specific mechanism. These limitations could be addressed in future research.

So, what are the clinical implications of our observations? EphA4 is highly expressed in adult brain and has important functions in regulating synaptic function. Increasing evidence found EphA4 linked to a range of diseases. In this study, our results suggested that the blockage of upregulated neuronal EphA4 reduced post-ischemia inflammatory injury through shifting microglia M2 polarization. Besides, our previous research also found blockage of EphA4/ephrinA3 signaling between neuron and astrocyte alleviated ischemic brain injury through preventing glutamate excitotoxicity [5]. Except for ischemia, EphA4 also plays important role in the pathogenesis of neurological disorders such as Alzheimer's disease (AD) [38], amyotrophic lateral sclerosis (ALS) [39] and glaucoma [32]. Therefore, we believe it is needed to elucidate the potential mechanisms by which neuronal EphA4 influence outcome of cerebral ischemia. This will also provide cues for clinical therapies of several neurological diseases.

In summary, our study demonstrated that neuronal EphA4 up-regulation after ischemia increased inflammatory injury through promoting microglia M1 polarization. The effects of EphA4 up-regulation on M1 polarization were attenuated by blocking NF-κB. Neuronal EphA4 silencing switched microglia from M1 to M2-phenotype, which in turn reduced inflammatory injury **(Graphical Abstract)**. We propose that EphA4 manipulation could be a promising therapeutic method for this kind of disease and develop clinical treatments for a variety of neurological diseases.

## Author contribution statement

Hui-Xing Wei; Jin-Shan Yang: Conceived and designed the experiments; Analyzed and interpreted the data; Wrote the paper.

Yun-Ni Guan; Ping-Ping Chen; Zhao-Zeng Rao: Performed the experiments; Contributed reagents, materials, analysis tools or data.

## Data availability statement

Data associated with this study has been deposited at jianguoyun; Shareable link: https://www.jianguoyun.com/p/Dawo4k8QgdndChicgYYFIAA.

## Funding

This work was partly supported by the 10.13039/501100001809National Natural Science Foundation of China to J-S Yang (Grant No.81901209) and the Joint Funds for the Innovation of Science and Technology, Fujian province to J-S Yang (Grant No. 2019Y9106) and the Training Project for Young and Middle-aged Core Talents in Health System of Fujian Province to H-X Wei (Grant No. 2019-ZQN-56) and 10.13039/501100003392Natural Science Foundation of Fujian Province to H-X Wei (Grant No. 2020J01958).

## Declaration of competing interest

The authors declare that they have no known competing financial interests or personal relationships that could have appeared to influence the work reported in this paper.
